# Plasmin-Induced Activation of Human Platelets Is Modulated by Thrombospondin-1, Bona Fide Misfolded Proteins and Thiol Isomerases

**DOI:** 10.3390/ijms21228851

**Published:** 2020-11-23

**Authors:** Claudia Pielsticker, Martin F. Brodde, Lisa Raum, Kerstin Jurk, Beate E. Kehrel

**Affiliations:** 1 Department of Anaesthesiology, Intensive Care and Pain Medicine, Experimental and Clinical Haemostasis, University of Muenster, 48149 Muenster, Germany; claudia_pielsticker@yahoo.de (C.P.); lisa.raum@outlook.de (L.R.); 2 OxProtect GmbH, 48149 Muenster, Germany; brodde@uni-muenster.de; 3 Center for Thrombosis and Hemostasis (CTH), University Medical Center of the Johannes Gutenberg-University Mainz, 55131 Mainz, Germany

**Keywords:** platelets, plasmin, thrombospondin-1, protein misfolding, thiol-isomerases

## Abstract

Inflammatory processes are triggered by the fibrinolytic enzyme plasmin. Tissue-type plasminogen activator, which cleaves plasminogen to plasmin, can be activated by the cross-β-structure of misfolded proteins. Misfolded protein aggregates also represent substrates for plasmin, promoting their degradation, and are potent platelet agonists. However, the regulation of plasmin-mediated platelet activation by misfolded proteins and vice versa is incompletely understood. In this study, we hypothesize that plasmin acts as potent agonist of human platelets in vitro after short-term incubation at room temperature, and that the response to thrombospondin-1 and the bona fide misfolded proteins Eap and SCN^−^-denatured IgG interfere with plasmin, thereby modulating platelet activation. Plasmin dose-dependently induced CD62P surface expression on, and binding of fibrinogen to, human platelets in the absence/presence of plasma and in citrated whole blood, as analyzed by flow cytometry. Thrombospondin-1 pre-incubated with plasmin enhanced these plasmin-induced platelet responses at low concentration and diminished them at higher dose. Platelet fibrinogen binding was dose-dependently induced by the C-terminal thrombospondin-1 peptide RFYVVMWK, Eap or NaSCN-treated IgG, but diminished in the presence of plasmin. Blocking enzymatically catalyzed thiol-isomerization decreased plasmin-induced platelet responses, suggesting that plasmin activates platelets in a thiol-dependent manner. Thrombospondin-1, depending on the concentration, may act as cofactor or inhibitor of plasmin-induced platelet activation, and plasmin blocks platelet activation induced by misfolded proteins and vice versa, which might be of clinical relevance.

## 1. Introduction

The tight regulation of platelet activity is a prerequisite to maintain vascular integrity in hemostasis and inflammation. Dysregulation of platelet function results in hyper- or hypo- activity, leading to thrombo-inflammatory or bleeding-associated complications. Platelet activity essentially regulates primary hemostasis but also coagulation, i.e., amplification of thrombin generation, and contributes importantly to immune and inflammatory responses [1,2]. However, the interaction of platelets with the fibrinolytic system is incompletely understood. The central fibrinolytic serine protease plasmin is generated through the cleavage of its zymogen Glu-plasminogen physiologically by the tissue-type (tPA), with fibrin as cofactor, and urokinase-type (uPA) plasminogen activators upon vascular injury and inflammation. Plasmin predominantly degrades fibrin within thrombi to fibrin degradation products (FDP) and D-dimer during clot resolution. Bound to fibrin and/or cell surfaces, plasmin is protected from its major inhibitors, α2-antiplasmin and α2-macroglobulin. Platelets exhibit pro- and anti- fibrinolytic properties, which were comprehensively reviewed by Colucci et al. [3]. In addition to direct binding to unstimulated and activated platelets, plasminogen binds to fibrin on thrombin-stimulated platelets [4,5]. Alternatively, the homotrimeric matricellular glycoprotein thrombospondin-1 (TSP-1) serves as cofactor for plasmin generation, forming a trimolecular complex with plasminogen and tPA [6,7]. TSP-1 is synthesized by megakaryocytes and stored as one of the most abundant α-granule protein in platelets [8]. In response to injury, TSP-1 is released via exocytosis of α-granules by activated platelets and rebound to the platelet surface via TSP-1 receptors, e.g., CD36, CD47, αIIbβ3, or indirectly, via immobilized fibrinogen or von Willebrand factor [9]. The multidomain structure of TSP-1 determines its multifunctional capacity to modulate differentially platelet functions dependent on the environmental conditions [10]. Besides TSP-1, activated platelets release the pro-fibrinolytic factors plasminogen and histidine-rich glycoprotein from α-granules, which also form a trimolecular complex together with TSP-1 on the platelet surface to amplify plasmin generation [11]. 

In response to injury, the misfolding of proteins occurs, which is characterized by protein unfolding of the native protein state, leading to the loss of physiological protein function. On the one hand, intermolecular β-sheet-rich structures, also termed “cross-β structures” [12], (stabilized by hydrogen bonding and hydrophobic interactions) are prominent features of misfolded proteins, tending to the formation of soluble amyloid-like oligomers and insoluble aggregated amyloid fibrils. Environmental changes regarding pH, temperature, oxidation, shear, glycation and denaturing charged surfaces present biological stressors, leading to protein amyloids [13,14]. On the other hand, non-amyloid amorphous protein aggregates are known to be induced by heat and chemical reduction. However, an increase in hydrophobicity is a common feature of all misfolded proteins compared to native ones. Misfolded proteins, including amyloids and amorphous aggregates, are recognized by tPA as cofactors through the interaction between the fibronectin type I domain of tPA and the cross-β structure of misfolded proteins to promote tPA-mediated conversion of plasminogen to plasmin [12,15]. Furthermore, plasmin has been implicated in the degradation of misfolded proteins, including amyloid-β, to facilitate their phagocytic clearance in concert with extracellular chaperones, thereby binding to exposed hydrophobic regions on misfolded proteins [16,17,18]. Soluble misfolded protein oligomers have been identified as agonists of human platelets such as oxidized low-density lipoprotein [19], amyloid-β peptide and glycated hemoglobin [20]. The amyloid-like cell binding domain with the amino acid sequence RFYVVMWK is localized within the globular C-terminus of TSP-1 [21], and corresponding peptides have been shown to agglutinate and activate platelets [22,23,24]. Previously, we showed that the virulence factor staphylococcal extracellular adherence protein (Eap), which is selectively secreted by *S. aureus*, and therefore, interacts with multiple host extracellular matrix proteins, shares amyloid-like properties and induces the activation of human platelets, which includes interactions with hydrophobic sites and the stimulation of surface-exposed thiol isomerases [25]. 

Platelets do not only contribute to plasmin generation; they also respond to plasmin. The effect of plasmin on platelets in vitro has been extensively investigated. However, previous studies provided evidence that plasmin exerts ambivalent effects on platelet function, thereby inducing/propagating or inhibiting platelet activation depending on the experimental conditions (for a review, see [26]). In this study, we hypothesize that plasmin acts as potent agonist of human platelets in vitro after short-term incubation at room temperature (RT), and that the response to injury glycoprotein TSP-1 and the bona fide misfolded proteins Eap and SCN^−^-denatured IgG interfere with plasmin, thereby modulating platelet activation. Therefore, we aimed to assess the activating effect of plasmin on human platelets in the absence and presence of plasma and in anticoagulated whole blood by flow cytometry, and its regulation by highly purified human TSP-1, by the C-terminal TSP-1 peptide RFYVVMWK and by surface exposed thiol isomerases. Further, we aimed to investigate the effect of plasmin on platelet activation induced by RFYVVMWK, Eap and NaSCN-treated IgG. 

## 2. Results

### 2.1. Plasmin Induces a-Granule Exocytosis and Adhesion Protein Binding of Human Platelets

**Figure 1:**
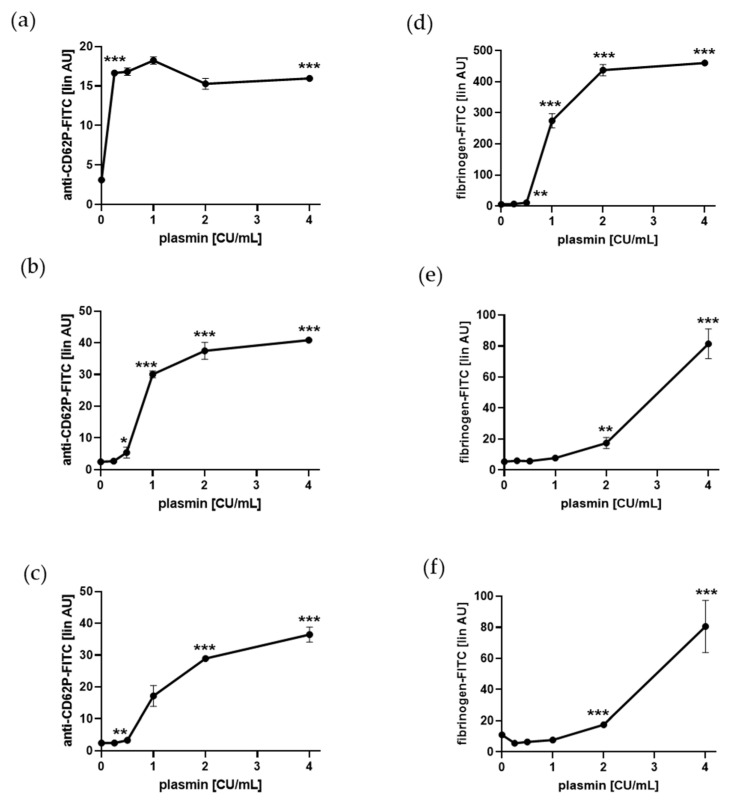
Plasmin induces platelet P-selectin surface expression and fibrinogen binding. Human platelets, gel-filtered (**a**,**d**), in PRP (**b**,**e**), in citrated whole blood (**c**,**f**) were treated with increasing concentrations of plasmin for 5 min at RT and labeled with anti-CD62P antibody (**a**–**c**) or fibrinogen-FITC (**d**–**f**). The linear median fluorescence intensity of gated platelets was analyzed and presented as arbitrary units (AU). Data are represented as means ± SD from three independent experiments. * *p* < 0.05, ** *p* < 0.01, *** *p* < 0.001 versus vehicle control without plasmin.

We used well-established and standardized protocols for the activation of human platelets for 5 min at RT (22 °C) in the absence (gel-filtered platelets) and presence (platelet-rich plasma, PRP) of plasma components and in citrated whole blood. We selected short-term plasmin incubation as well as RT, and not 37 °C, for our experiments to analyze more proteolytic independent properties of plasmin on platelet activation. For all conditions, we observed a dose-dependent increase in P-selectin (CD62P) surface presentation as marker for α-granule exocytosis and binding of FITC-conjugated fibrinogen to plasmin-treated platelets as assessed by flow cytometry (Figure 1). Even 0.25 CU/mL plasmin induced a significant and saturable increase in P-selectin expression of gel-filtered platelets, whereas in the presence of plasma (PRP) and in whole blood, 0.5 CU/mL plasmin was sufficient for a significant increase (Figure 1a–c). Significant fibrinogen binding was achieved with 0.5 CU/mL plasmin for gel-filtered platelets, whereas 2 CU/mL plasmin were needed in PRP and whole blood (Figure 1d–f). Interestingly, significant binding of FITC-conjugated human TSP-1 to platelets, gel-filtered and in PRP, was even induced by lower plasmin concentrations, 0.25 CU/mL and 1.0 CU/mL plasmin, respectively (Supplementary Materials Figure S1a,b). 

These data clearly demonstrate that short-term treatment with low and high concentrations of plasmin at RT mediate platelet activation, i.e., α-granule secretion and binding of fibrinogen and TSP-1, in vitro. 

### 2.2. TSP-1 Acts As a Promoter and Inhibitor of Plasmin-Induced Platelet α-Granule Exocytosis

**Figure 2:**
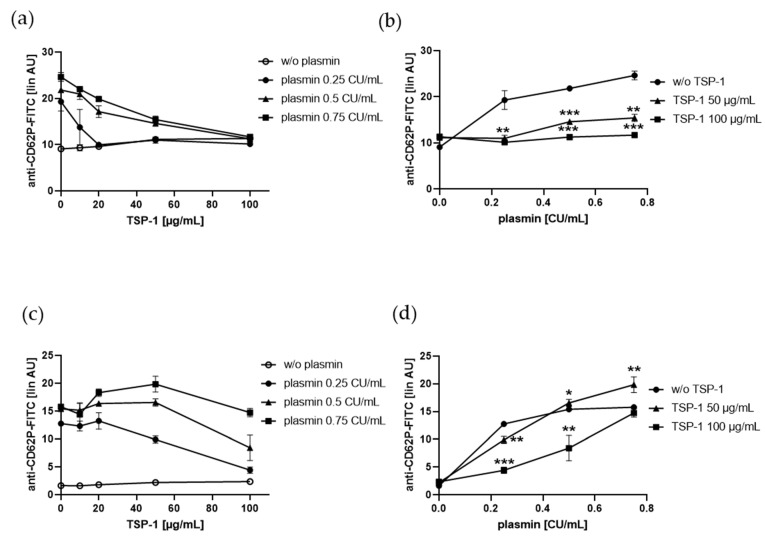
Effect of TSP-1 on plasmin-induced P-selectin (CD62P) expression on human platelets. (**a**,**b**) Human gel-filtered platelets were treated with vehicle control or increasing concentrations of plasmin in the absence or presence of increasing concentrations of human TSP-1 for 5 min at RT before fixation and staining with anti-CD62P antibody. (**c**,**d**) Plasmin was pre-incubated with TSP-1 for 30 min at 4 °C and gel-filtered platelets were treated with plasmin-bound TSP-1 for 5 min at RT before fixation and staining with anti-CD62P antibody. (**a**,**c**) Platelet CD62P-surface expression induced by plasmin depending on increasing concentrations of added TSP-1. (**b**,**d**) Effect of increasing concentrations of plasmin on platelet CD62P surface expression in the presence of added TSP-1 (50 µg/mL, 100 µg/mL). The linear median fluorescence intensity of gated platelets was analyzed and presented as arbitrary units (AU). Data are represented as means ± SD from three independent experiments. * *p* < 0.05, ** *p* < 0.01, *** *p* < 0.001 versus corresponding plasmin concentration without (w/o) TSP-1.

In this study, we showed that plasmin induces the binding of TSP-1 to human platelets. TSP-1 has been reported to propagate aggregation of human platelets induced by collagen via binding to the scavenger receptor CD36 [27], and in mice, TSP-1 supports thrombus stabilization on collagen [28]. Further, we previously identified TSP-1 as high shear substrate for human platelets [29]. To elucidate the effect of TSP-1 on plasmin-induced platelet activation, we incubated gel-filtered platelets simultaneously with increasing concentrations of plasmin and TSP-1. TSP-1 alone, even at a concentration of 100 µg/mL, did not induce platelet P-selectin surface expression. However, it diminished plasmin-induced P-selectin expression in a dose-dependent manner. Additionally, 100 µg/mL TSP-1 even prevented this platelet response when plasmin was used up to 0.75 CU/mL (Figure 2a,b). Interestingly, when plasmin was pre-incubated with TSP-1 for 30 min at 4 °C, P-selectin exposure was increased in the presence of low TSP-1 concentrations (20, 50 µg/mL), but inhibited at high TSP-1 concentration (50, 100 µg/mL), dependent on the used plasmin dose (Figure 2c,d). 

**Figure 3:**
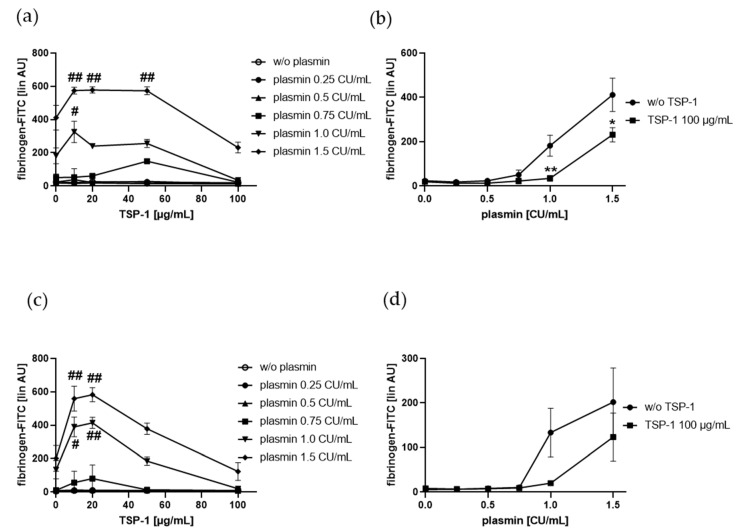
Effect of TSP-1 on plasmin-induced fibrinogen binding to human platelets. (**a**,**b**) Human gel-filtered platelets plus fibrinogen-FITC were treated with vehicle control or increasing concentrations of plasmin in the absence or presence of increasing concentrations of added human TSP-1 for 5 min at RT before fixation. (**c**,**d**) Plasmin was pre-incubated with TSP-1 for 30 min at 4 °C and gel-filtered platelets plus fibrinogen-FITC were treated with plasmin-bound TSP-1 for 5 min at RT before fixation. (**a**,**c**) Platelet fibrinogen binding induced by plasmin depending on increasing concentrations of TSP-1. (**b**,**d**) Effect of increasing concentrations of plasmin on platelet fibrinogen binding in the presence of added TSP-1 (100 µg/mL). The linear median fluorescence intensity of gated platelets was analyzed and presented as arbitrary units (AU). Data are represented as means ± SD from three independent experiments. # *p* < 0.05, ## *p* < 0.01 versus corresponding plasmin concentration without (w/o) TSP-1; * *p* < 0.05, ** *p* < 0.01 versus corresponding plasmin concentration without (w/o) TSP-1.

These results indicate that TSP-1 may act as promoter or inhibitor of plasmin-induced α-granule exocytosis in vitro, depending on its concentration. Furthermore, we observed similar effects of TSP-1 on binding of fibrinogen to human gel-filtered platelets treated with plasmin (Figure 3), demonstrating that TSP-1 exerts ambivalent effects not only on platelet α-granule exocytosis, but also on the activation of the platelet integrin αIIbβ3, leading to binding of soluble fibrinogen. 

### 2.3. Plasmin-Induced Fibrinogen Binding to Human Platelets Is Diminished by the C-Terminal TSP-1 Peptide RFYVVMWK, and Plasmin Reduced RFYVVMWK-Induced Fibrinogen Binding Vice Versa

Although native TSP-1 alone does not induce platelet activation, it has been suggested that conformational changes in TSP-1 are triggered by local intravascular alterations or proteolytic cleavage, leading to the exposure of distinct domains, e.g., the C-terminal cell binding domain with the amino acid sequence RFYVVMWK [10,30]. Corresponding synthetic peptides are well-known to mediate platelet agglutination via binding to the integrin associated protein (CD47), as well as platelet aggregation in a FcRγ-chain dependent manner [23,24]. To test whether this TSP-1 peptide triggered tPA-mediated generation of plasmin via its potential amyloid-like properties [21], RFYVVMWK was pre-incubated with recombinant human tPA for 20 min at 4 °C and tPA-dependent conversion of plasminogen to plasmin was determined in a colorimetric assay. RFYVVMWK dose-dependently increased the generation of plasmin with an optimum at 20 µM, as detected by the increase of absorbance at 405 nm, which was caused by the cleavage of a colorimetric plasmin-specific substrate. In comparison, a higher concentration of pre-incubated RFYVVMWK (50 µM, 100 µM) led to decreased generation of plasmin, and even to further inhibition compared to tPA alone (Supplementary Materials Figure S2a,b). 

**Figure 4:**
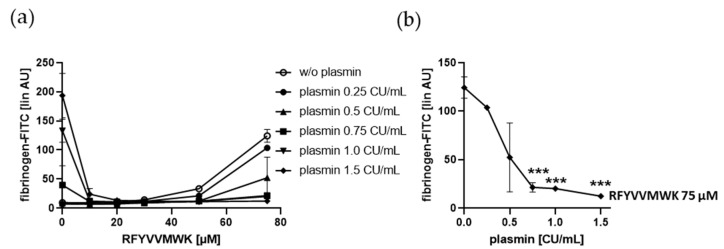
Effect of C-terminal TSP-1 peptide RFYVVMWK on plasmin-induced fibrinogen binding to human platelets. Human gel-filtered platelets plus fibrinogen-FITC were treated with vehicle control or increasing concentrations of plasmin in the absence or presence of increasing concentrations of RFYVVMWK for 5 min at RT before fixation. (**a**) Platelet fibrinogen binding induced by plasmin depending on increasing concentrations of RFYVVMWK. (**b**) Effect of increasing concentrations of plasmin on platelet fibrinogen binding in the presence of RFYVVMWK (75 µM). The linear median fluorescence intensity of gated platelets was analyzed and presented as arbitrary units (AU). Data are represented as means ± SD from three independent experiments. *** *p* < 0.001 versus without (w/o) plasmin.

When plasmin and RFYVVMWK were added simultaneously to gel-filtered platelets, plasmin-induced platelet fibrinogen binding was dose-dependently diminished by the TSP-1 peptide in the range of 0.25 and 1.5 CU/mL plasmin and up to 30 µM RFYVVMWK (Figure 4a). It is important to note that a peptide concentration of 50 µM and higher alone significantly induced the binding of fibrinogen to the surface of gel-filtered platelets. Fibrinogen binding induced by 50 µM and 75 µM RFYVVMWK was dose-dependently reduced by increasing plasmin concentrations up to 1.5 CU/mL plasmin (Figure 4a,b).

Based on these results, we suggest that the C-terminal TSP-1 peptide RFYVVMWK interferes with plasmin-induced platelet fibrinogen binding and vice versa.

### 2.4. Fibrinogen Binding to Human Platelets Induced by the Bona Fide Amyloid-Like Eap or NaSCN-Modified IgG is Diminshed by Plasmin at Low Doses

**Figure 5:**
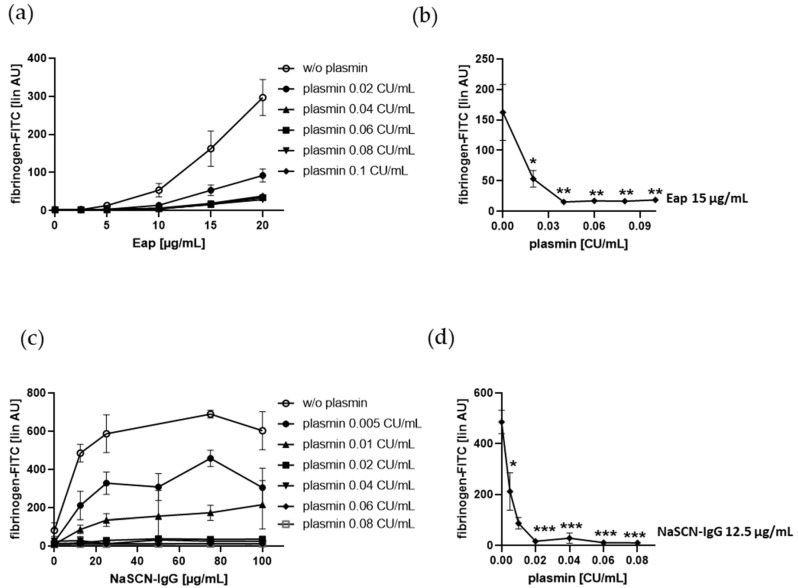
Effect of Eap and NaSCN-IgG on plasmin-induced fibrinogen binding to human platelets. Human gel-filtered platelets plus fibrinogen-FITC were treated with vehicle control or increasing concentrations of plasmin in the absence or presence of (**a**,**b**) Eap or (**c**,**d**) NaSCN-IgG for 5 min at RT before fixation. (**a**,**c**) Platelet fibrinogen binding induced by plasmin depending on increasing concentrations of Eap and NaSCN-IgG, respectively. (**b**,**d**) Effect of increasing concentrations of plasmin on platelet fibrinogen binding induced by Eap (15 µg/mL) or NaSCN-IgG (12.5 µg/mL). The linear median fluorescence intensity of gated platelets was analyzed and presented as arbitrary units (AU). Data are represented as means ± SD from three independent experiments. * *p* < 0.05, ** *p* < 0.01, *** *p* < 0.001 versus without (w/o) plasmin.

Eap-induced fibrinogen binding to gel-filtered platelets was dose-dependently decreased by plasmin up to 0.1 CU/mL plasmin (Figure 5a,b). We also tested purified IgG treated with the chaotropic and protein denaturing agent, NaSCN, and showed that this modified IgG (8–18 mg/mL in human plasma) also mediated platelet fibrinogen binding dose-dependently. Here, even 0.02 CU/mL plasmin completely inhibited this platelet response induced by up to 100 µg/mL NaSCN-IgG (Figure 5c,d). These data indicate that plasmin inhibits platelet fibrinogen binding induced by the bona fide misfolded protein Eap and SCN^−^-denatured IgG.

Using again the colorimetric tPA-mediated plasminogen activation assay, we demonstrated that Eap, as well as NaSCN-denatured IgG, modulates tPA-induced plasmin generation. Pre-incubation of tPA with Eap resulted in an up to five-fold increase in absorbance at 405 nm with an optimum at 0.1 µg/mL, whereas higher concentrations prevented the triggering effect of Eap (Supplementary Materials Figure S3a,b). NaSCN-denatured IgG dose-dependently enhanced the conversion of plasminogen to plasmin by tPA up to 10 µg/mL (Supplementary Materials Figure S4a,b). These results support the functional impact of Eap and NaSCN-treated IgG as misfolded proteins, not only on platelets, but also on tPA-induced plasminogen activation in vitro. 

### 2.5. Plasmin-Induced Platelet P-selectin Expression and Fibrinogen Binding Depend on Enzymatically Catalyzed Disulfide Exchange on the Platelet Surface

**Figure 6:**
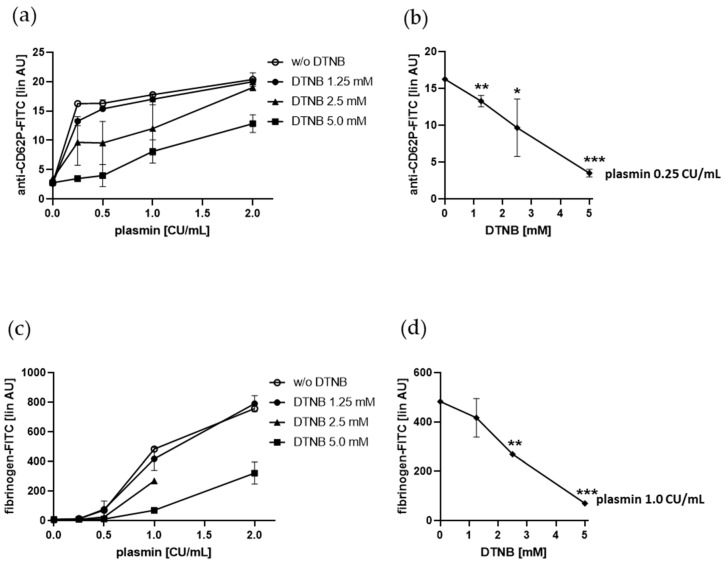
Effect of DTNB on plasmin-induced platelet P-selectin (CD62P) surface expression and fibrinogen binding. (**a**,**b**) Human gel-filtered platelets were pre-incubated with DTNB for 5 min at RT and treated with vehicle control or increasing concentrations of plasmin for 5 min at RT before fixation and staining with anti-CD62P antibody. (**c**,**d**) Human gel-filtered platelets were pre-incubated with DTNB for 5 min at RT and treated with vehicle control or increasing concentrations of plasmin in the presence of fibrinogen-FITC for 5 min at RT before fixation. (**a**,**c**) Effect of DTNB on platelet CD62P surface expression and fibrinogen binding induced by increasing concentrations of plasmin. (**b**,**d**) Effect of DTNB on platelet CD62P surface expression and fibrinogen binding induced by 0.25 CU/mL and 1.0 CU/mL plasmin, respectively. The linear median fluorescence intensity of gated platelets was analyzed and presented as arbitrary units (AU). Data are represented as means ± SD from three independent experiments. * *p* < 0.05, ** *p* < 0.01, *** *p* < 0.001 versus without (w/o) DTNB.

As we had determined that Eap mediated platelet activation via surface exposed thiol isomerases [25], we tested whether extracellular free thiols and enzymatic disulfide exchange were involved in plasmin-induced platelet activation. We pre-incubated gel-filtered platelets with the thiol-reactive and cell-impermeable compound DTNB to block free protein thiols on the platelet surface prior treatment with plasmin. DTNB diminished plasmin-induced platelet P-selectin surface expression as well as fibrinogen binding in a dose dependent manner, leading to complete inhibition by 5 mM DTNB, when platelets were treated with 0.25 CU/mL and 1.0 CU/mL plasmin, respectively (Figure 6).

From these results, we conclude that surface exposed free thiols are crucial for plasmin-induced platelet activation.

**Figure 7:**
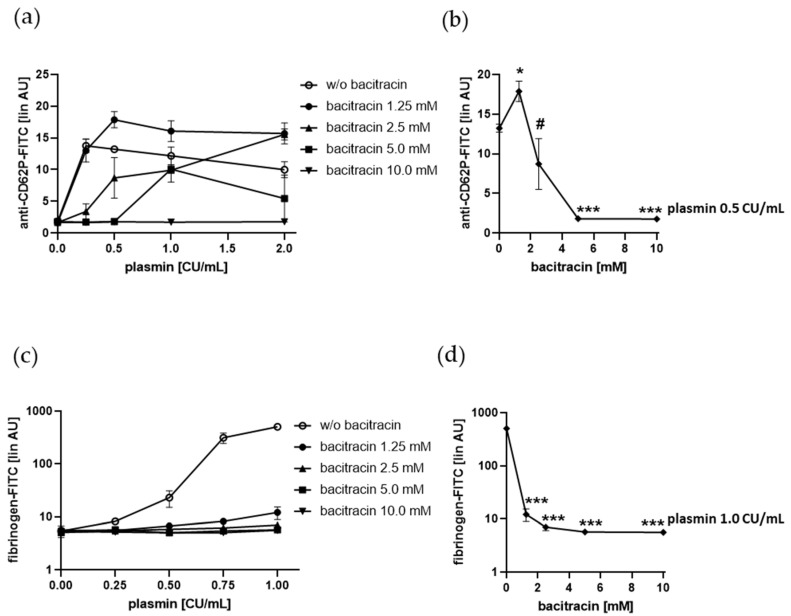
Effect of bacitracin on plasmin-induced platelet P-selectin (CD62P) surface expression and fibrinogen binding. (**a**,**b**) Human gel-filtered platelets were pre-incubated with bacitracin for 5 min at RT and treated with vehicle control or increasing concentrations of plasmin for 5 min at RT before fixation and staining with anti-CD62P antibody. (**c**,**d**) Human gel-filtered platelets were pre-incubated with bacitracin for 5 min at RT and treated with vehicle control or increasing concentrations of plasmin in the presence of fibrinogen-FITC for 5 min at RT before fixation. (**a**,**c**) Effect of bacitracin on platelet CD62P surface expression and fibrinogen binding induced by increasing concentrations of plasmin. (**b**,**d**) Effect of bacitracin on platelet CD62P surface expression and fibrinogen binding induced by 0.5 CU/mL and 1.0 CU/mL plasmin, respectively. The linear median fluorescence intensity of gated platelets was analyzed and presented as arbitrary units (AU). Data are represented as means ± SD from three independent experiments. * *p* < 0.05, *** *p* < 0.001 versus without (w/o) bacitracin; # *p* < 0.05 versus plasmin 0.5 CU/mL in the presence of 1.25 mM bacitracin.

Previously, we showed that enzymatically catalyzed exchange of disulfide bonds on the surface of human platelets is essential for sustained αIIbβ3 integrin ligation, and is involved in granule secretion [31]. Here, we observed that the endoplasmic reticulum-associated chaperone and thiol isomerase protein disulfide isomerase (PDI) were crucially involved. Surface exposed PDI also plays a role in the thiol-dependent recruitment of distinct coagulation factors to thrombin-stimulated platelets [32]. Using the cell-impermeable thiol-isomerase inhibitor bacitracin, we observed that 1 mM bacitracin increased, but higher bacitracin concentrations diminished, plasmin-induced platelet P-selectin surface expression (Figure 7a,b) as well as fibrinogen binding (Figure 7c,d). Bacitracin at a concentration of 5 mM completely prevented P-selectin surface exposure and fibrinogen binding when gel-filtered platelets were treated with 0.5 CU/mL and 1.0 CU/mL plasmin, respectively. 

These data indicate that surface exposed thiol-isomerases are importantly involved in plasmin-induced platelet activation.

## 3. Discussion

In this study, we established plasmin as a potent agonist of human platelets in vitro, inducing P-selectin surface expression and binding of fibrinogen, as well as TSP-1 at low and high plasmin concentrations, in the absence and presence of plasma and in citrated whole blood. Furthermore, we identified human TSP-1 as cofactor and inhibitor of plasmin-induced platelet activation, depending on its concentration, when bound to plasmin. Plasmin inhibited platelet fibrinogen binding induced by the misfolded soluble proteins C-terminal TSP-1 peptide RFYVVMWK, Eap and denatured IgG, which also trigger tPA-mediated conversion of plasminogen to plasmin. Our data suggest that plasmin-induced platelet activation depends on extracellular thiol isomerase-dependent disulfide exchange. Based on these results, we conclude that the interaction between plasmin and the response to injury protein TSP-1, Eap and denatured IgG might be important in the differential modulation of platelet activation especially under inflammatory conditions.

Early studies already reported that plasmin exerts differential effects on human platelets in vitro, dependent on experimental temperature, incubation time and presence of plasma proteins. When experiments were performed at 37 °C, it was shown that only higher plasmin concentrations (>1 CU/mL) were able to significantly induce shape change, δ-granule release and aggregation of washed and gel-filtered platelets at 37 °C [33,34,35]. Interestingly, lower plasmin concentrations (<1 CU/mL), even with 0.2 CU/mL, efficiently mediated platelet aggregation at RT (22 °C), but also the activation of integrin αIIbβ3, release of α-, δ-granules and lysosomes when plasmin was incubated for 5 min [36,37]. In our study, we used human platelets isolated by gel-filtration and confirmed that plasmin incubation for 5 min at RT (22 °C) induces P-selectin surface expression as marker for α-granule exocytosis, as well as fibrinogen binding at low and high plasmin doses, in a concentration-dependent manner. We observed these platelet activating effects not only in the absence of plasma factors, but also in diluted platelet-rich plasma and citrated whole blood, where 0.5 CU/mL of plasmin still significantly increased P-selectin on the platelet surface despite the presence of the plasmatic plasmin inhibitors α2-antiplasmin and α2-macroglobulin. Blockmans et al. reported the aggregatory capacity of plasmin on gel-filtered platelets, but no aggregation response in PRP, and suggested that in PRP, the α2-antiplasmin-mediated inhibition of plasmin activity might be responsible for this observation [35]. This was in line with studies by Kunapuli’s group which demonstrated that the serine protease inhibitor PMSF at mM concentration prevents the aggregation of washed platelets induced by 1 CU/mL of plasmin [38]. However, Cramer et al. showed that plasmin in the presence of plasma degrades integrin αIIbβ3 [39], and pre-incubation of platelets with lower plasmin concentrations (<1 CU/mL) leads to the inhibition of platelet aggregation in response to other platelet agonists such as thrombin, collagen and Ca^2+^-ionophore A23187 [40], implicating that the proteolytic activity of plasmin also diminished platelet activation. Selim et al. demonstrated inhibition of plasmin-induced platelet aggregation by high- and low-molecular-mass kininogens by interfering with the Kringle 5 domain, but not with the active site of plasmin [41]. 

These and our observations that plasmin is able to activate platelets, especially at lower temperature and in the presence of plasmin inhibitors in plasma, indicate that the proteolytic activity of plasmin is not an essential requirement for its activating action on human platelets. Furthermore, these results might have clinical and therapeutic impact under conditions when fibrinolytic activity and platelet activation are increased, combined with hypothermia, as observed for patients with trauma and during cardiopulmonary bypass surgery [42]. 

The matricellular glycoprotein TSP-1, which is predominantly secreted by activated platelets, monocytes, neutrophils and vascular cells in response to injury, has been recognized as modulator of plasmin activation and activity. In vitro binding assays demonstrated that isolated human TSP-1 forms a bimolecular complex with plasminogen and a trimolecular complex with plasminogen and histidine-rich glycoprotein to facilitate tPA-mediated plasminogen conversion to plasmin [6,11]. Similar to plasminogen-tPA-fibrin complexes, plasmin in complex with TSP-1 is protected from inactivation by α2-antiplasmin [43]. Thus, TSP-1 may act as a promoter of fibrinolysis. On the other hand, TSP-1 in solution binds tightly to plasmin and acts as a slow inhibitor of plasmin activity with a stoichiometry of one mol plasmin to one mol TSP-1 trimer [44,45]. The lysine-binding Kringle domains of plasmin have been implicated in this functional interaction, as TSP-1 is not able to inhibit plasmin when it is complexed with streptokinase or ε-aminocaproic acid [45]. Here, we showed for the first time that highly purified human TSP-1 decreases dose-dependently plasmin-induced platelet P-selectin surface expression and fibrinogen binding when platelets were treated for 5 min at RT. This was also observed when TSP-1 was pre-incubated with plasmin in a molar ratio of about 1:1 (e.g., 100 µg/mL/222 nM TSP-1 vs. 0.5 CU/mL/250 nM plasmin) prior platelet treatment, suggesting that TSP-1 might serve as competitive inhibitor for plasmin-induced platelet activation. Conversely, TSP-1 also enhanced plasmin-mediated effects on platelets when pre-incubated with plasmin prior platelet treatment for 30 min in a molar ratio of <1 mol of trimeric TSP-1 to 1 mol of plasmin. This observation might be explained by the excess of plasmin in relation to TSP-1, leading to plasmin-mediated cleavage fragments of TSP-1 such as the C-terminal fragment that includes the platelet-activating amino acid sequence RFYVVMWK [22,23,24]. To prove this, future studies are necessary to show that plasmin may cleave TSP-1 in a similar way as observed for the neutrophil serine protease elastase [30]. Interestingly, our data revealed that non-platelet activating concentrations of the TSP-1 C-terminal peptide RFYVVMWK competitively inhibits plasmin-induced fibrinogen binding to platelets and that plasmin competitively inhibits RFYVVMWK-induced platelet fibrinogen binding, too. From these results it is very likely that the interaction between TSP-1 and plasmin strongly influences the effect of plasmin on human platelets. 

As shown by others and us in this study, RFYVVMWK induces platelet activation and aggregation, and it has been suggested that signaling via the FcRγ-chain may be involved. However, it could be not proved so far that the platelet receptor for this TSP-1 peptide, CD47, is involved in this pathway [23]. Notably, McDonald et al. demonstrated the amyloid-like properties of the recombinantly expressed C-terminal domain of TSP-1, which is enriched in β-sheet secondary and hydrophobic structures based on two sequences including VVM motifs [21]. Others already published that amyloids or proteins with amyloid-like properties are able to activate platelets [19,20]. These results are in line with our data, demonstrating that also the natural *S. aureus* adhesin Eap with amyloid-like properties [25], and SCN^−^-denatured IgG, which is likely to be generated by enhanced catabolism of cyanides and tobacco products, induced platelet fibrinogen binding. Thus, all these misfolded proteins may share similar mechanisms to activate platelets. It is important to note that plasmin in concentrations that do not activate platelets competitively inhibited platelet fibrinogen binding induced by Eap and SCN^−^-denatured IgG, too. Based on these data we conclude that very low concentrations of plasmin may protect platelets from platelet activation by misfolded proteins and vice versa.

Misfolded proteins, especially amyloids with cross-β structure, have been shown to act as cofactors for tPA in plasminogen conversion to plasmin [46,47,48], and the fibronectin type I domain of tPA has been identified as binding site for amyloids [49]. In this study, we validated the tPA cofactor function of the C-terminal TSP-1 peptide RFYVVMWK, Eap and NaSCN-denatured IgG in a colorimetric plasminogen activation assay, suggesting that the functional impact of these proteins on tPA activation as well as on modulation of platelet function and interference with plasmin-induced platelet activation might be explained by their misfolded properties. 

We further showed that Eap not only activate platelets, but also increases platelet surface thiol-isomerase activity, and that Eap-mediated platelet activation is inhibited by the thiol-reactive compound DTNB and the extracellular thiol isomerase inhibitor bacitracin [25]. Similarly, our results indicated that platelet P-selectin surface expression and fibrinogen binding induced by plasmin depends on disulfide thiol isomerase activity on the platelet surface, suggesting a crucial role of enzymatically catalyzed disulfide exchange for platelet activation mediated by misfolded proteins and plasmin. 

Our observation that plasmin as well as misfolded proteins mediate a tremendous increase in binding of external added FITC-conjugated fibrinogen might be explained on the one hand by direct receptor interaction and activation of the integrin αIIbβ3 via inside-out signaling. Kunapuli’s group showed that plasmin mediates activation of PAR-4 at the thrombin cleavage site [38], but intracellular signaling has not been addressed so far. Herczenik et al. provided evidence that different amyloidic proteins, also as soluble forms, activates human platelets in part via GPIba of the major von Willebrand factor receptor complex GPIb-V-IX, via the scavenger receptor CD36 and through MAP kinase p38 and cyclocoxygenase-1 dependent signaling [20]. In line with this study, we observed that fibrin, which has also been proposed to exhibit amyloid-like properties [50], mediates activation and thrombin generation via GPIbα and CD36 in a src-family kinase and Syk dependent manner [51]. On the other hand, we previously identified α-defensins, which are secreted by activated neutrophils, to form polymeric fibrinogen and TSP-1 amyloid-like structures, which bound to platelets via integrin αIIbβ3, thereby contributing to α-defensin-mediated platelet activation via outside-in signaling [52]. A clear limitation of this study is that we did not focus on specific platelet receptors and signaling pathways that might be involved in plasmin-mediated platelet activation and in its regulation by TSP-1 and misfolded proteins. These important aspects and the likely misfolded properties of plasmin have to be explored in future studies. 

In summary, we established plasmin as potent platelet agonist after short-term treatment at room temperature, which induces α-granule release and binding of fibrinogen and TSP-1 in a thiol isomerase-dependent manner, even in the presence of plasma factors. Our data suggest potential competitiveness between plasmin, TSP-1 and the bona fide misfolded proteins Eap and SCN^−^-denatured IgG, leading to a reduced platelet activating potential of plasmin and misfolded proteins, when the molar ratios are balanced. This process might be protectively relevant under physiological conditions of inflammation. Furthermore, this mechanism might be of clinical relevance and should be considered for thrombolytic therapy of thrombo-inflammatory diseases, when plasmin activity is increased and when the blood temperature is decreased.

## 4. Materials and Methods 

### 4.1. Blood Collection 

Citrate-anticoagulated whole blood (10.6 mmol/L final concentration of trisodium citrate solution) was collected by puncture of the antecubital vein from healthy volunteers (females, males from Germany between 25 and 37 years of age) who did not take any medication for 14 days. The health status was assessed by trained physicians based on medical history, physical examination, vital signs and routine laboratory data from the local medical office of the University of Muenster. All donors gave their informed consent to participate in the study. The study was conducted in accordance with the Declaration of Helsinki, and the protocol was approved by the local Ethics Committee of the University of Muenster; 2010-188-f-S.

### 4.2. Preparation of Human Platelets 

Platelet activation studies were conducted within 1 h after blood collection. For studies in platelet-rich plasma (PRP) citrate-anticoagulated whole blood was centrifuged at 200× *g* for 10 min at room temperature (RT). Gel-filtered platelets were generated as described with slight modifications [53]. Platelets in PRP was gel-filtered on Sephadex CL-2B, which was equilibrated with Tyrode’s buffer (140 mM NaCl, 2.7 mM KCl, 0.42 mM NaH_2_PO_4_, 12 mM NaHCO_3_, 5 mM glucose, 5.5 mM HEPES, pH 7.4) containing 1% bovine serum albumin (Sigma-Aldrich, St. Louis, MO, USA) and 0.1 U/mL apyrase (Sigma-Aldrich).

### 4.3. Preparation of Denatured Human IgG by NaSCN Treatment

Human Immunoglobulin G (Gammagard^®^ S/D, Baxter, Unterschleißheim, Germany) was incubated with 2 M NaSCN for 24 h at RT. Denatured IgG-NaSCN was isolated and desalted with Hanks‘ Balanced Salt Solution, pH 7.4 (GIBCO^®^ HBSS, Invitrogen, Darmstadt, Germany) using Sephadex G-25 PD-10 coloumns (Amersham Biosciences, Freiburg, Germany). 

### 4.4. Analysis of P-selectin (CD62P) Expression on Isolated Platelets by Flow Cytometry

Platelets in PRP or gel-filtered were adjusted to 5 × 10^7^ platelets/mL with Tyrode’s buffer and incubated with human plasmin (Kordia, Leiden, The Netherlands), the C-terminal TSP-1 peptide RFYVVMWK (Bachem Biochemica GmbH, Weil am Rhein, Germany) for 5 min at RT (22 °C). Recombinant Eap [54] or IgG-NaSCN was incubated with gel-filtered platelets for 35 min at RT in the presence of 2 mM CaCl_2_. Dithiobisnitrobenzoic acid (DTNB, Sigma-Aldrich) or bacitracin (Sigma Aldrich) were pre-incubated with platelets for 5 min at RT prior to activation. In some experiments highly purified human TSP-1 [29] was immediately added to platelets prior incubation with plasmin or pre-incubated with plasmin for 30 min at 4 °C. Platelets were fixed with 0.5% formaldehyde in Tyrode’s buffer (final concentration), washed and incubated with predefined saturating concentrations of FITC-conjugated mouse anti-human CD62P antibody (5 µg/mL of clone AK-4, Becton Dickinson Biosciences, Heidelberg, Germany) as described [55]. Platelet labeling with 5 µg/mL of FITC-conjugated IgG_1_ isotype control (Becton Dickinson Biosciences) served as negative control to distinguish between nonspecific IgG_1_ and anti-CD62P antibody binding. Washed platelet samples were analyzed by flow cytometry (FACSCalibur, Becton Dickinson Biosciences) using the CellQuest-Pro Software (maximum excitation wavelength of argon laser 488 nm, emission maximum 520 nm). Forward versus side scatter gating was used to discriminate single platelets. The median of fluorescence intensity (FL-1), presented by the CellQuest-Pro Software (version 5.1; Becton Dickinson Biosciences) as linear arbitrary units (AU), which were automatically calculated from logarithmic channel values, of a total of 5000 events were analyzed for each sample.

### 4.5. Analysis of P-selectin Expression on Platelets in Whole Blood by Flow Cytometry

Citrated whole blood was incubated with different concentrations of plasmin for 5 min at RT and fixed with 0.2% formaldehyde in Tyrode’s buffer (final concentration) as described [56]. Fixed platelets were stained with 5 µg/mL mouse anti-human CD42a-PE antibody (Becton Dickinson Biosciences) for 45 min at RT (22 °C) and lysis of erythrocytes was performed according to the manufacturer’s instruction (Beckman Coulter, Krefeld, Germany) [56]. Platelets were gated as CD42a positive cells compared to CD42a negative blood cells (e.g., erythrocytes or leukocytes) in combination with forward versus side scatter gating. The median of fluorescence intensity (FL-1), presented by the CellQuest-Pro Software (version 5.1; Becton Dickinson Biosciences) as linear arbitrary units (AU), which were automatically calculated from logarithmic channel values, of a total of 5000 events were analyzed for each sample. 

### 4.6. Flow Cytometric Analysis of Exogenous Fibrinogen and Thrombospondin-1 Binding to Platelets

Highly purified human fibrinogen (Enzyme Research Laboratories, South Bend, IN, USA) and TSP-1 [29] were conjugated with fluorescein isothiocyanate (FITC) via FITC-celite (Sigma-Aldrich) according to Xia et al. [57]. Binding of FITC-coupled human fibrinogen and TSP-1 to platelets, isolated and in citrated whole blood, was performed and analyzed as described [25,31]. Briefly, in the presence of 150 µg/mL fibrinogen-FITC or 50 µg/mL TSP-1-FITC, whole blood, PRP or gel-filtered platelets were incubated with plasmin or bona fide misfolded proteins/peptide and fixed as described above. The median fluorescence intensity of unspecific binding of FITC-fibrinogen and FITC-TSP-1 to platelets was calculated when gel-filtered platelets were pre-incubated with an excess concentration of unlabeled fibrinogen (500 µg/mL) or TSP-1 (250 µg/mL) and served as cut-off for specific platelet binding of FITC-conjugated fibrinogen and TSP-1. The median of fluorescence intensity (FL-1), presented by the CellQuest-Pro Software (version 5.1; Becton Dickinson Biosciences) as linear arbitrary units (AU), which were automatically calculated from logarithmic channel values, of a total of 5000 events were analyzed for each sample. 

### 4.7. t-PA-dependent Plasminogen Conversion to Plasmin

A colorimetric plasminogen activation assay was used to test the ability of the bona fide misfolded proteins RFYVVMWK, Eap and NaSCN-treated IgG for affecting tPA activity. Using 96-well polystyrene plates (Nunc, Roskilde, Denmark), human recombinant tPA (final concentration 0.5 µg/mL Actilyse, Boehringer-Ingelheim, Ingelheim, Germany) was pre-incubated with different concentrations of misfolded proteins for 20 min at 4 °C in PBS, pH 7.4. The reaction was started by the addition of 50 µg/mL plasminogen (Sigma-Aldrich) and 600 µM of the plasmin-specific substrate l-pyroglutamyl-l-phenylalanyl-l-lysine-p-nitroaniline hydrochloride (S-2403™, Chromogenix, Milano, Italy). tPA-mediated conversion of plasminogen to plasmin, was recorded by the change in absorbance at 405 nm on a MRX microplate reader (Dynatech, Denkendorf, Germany) up to 90 min with 10 min intervals at 37 °C based on the color development of the cleaved substrate S-2403 by generated plasmin. 

### 4.8. Data Analysis and Statistics

Data are presented as mean ± standard deviation (SD). GraphPad Prism software (version 6.07 for Windows, GraphPad Software, La Jolla, CA, USA) was used for data presentation and statistical analysis. In case of normal distribution parametric tests were performed for comparisons between two groups by two-tailed Student’s *t*-test, for comparisons between > 2 groups by one-way or two-way analysis of variance (ANOVA) followed by Tukey’s multiple comparisons test. For comparisons of each of a number of treatment groups with a single control group, one-way ANOVA followed by Dunnett post hoc test was performed. For comparison of nonparametric data sets the Mann-Whitney *U* test (comparison of 2 groups) or the Kruskal-Wallis test (comparison of >2 groups) followed by Dunn post hoc test for multiple comparisons was used. *p* < 0.05 was considered statistically significant.

## 5. Patents

M.F.B. and B.E.K. hold a patent on the recognition and removal of misfolded proteins. This patent is not based on, nor influenced by, the research presented in this manuscript (US-Patent number: 10416170).

## Supplementary Materials

The following are available online at https://www.mdpi.com/1422-0067/21/22/8851/s1.

## Abbreviations

AU	arbitrary units
DTNB	5,5’-dithiobis-(2-nitrobenzoic acid)
Eap	extracellular adherence protein
FDP	fibrin degradation products
PDI	protein disulfide isomerase
PRP	platelet-rich plasma
RT	room temperature
tPA	tissue-type plasminogen activator
TSP-1	thrombospondin-1
uPA	urokinase-type plasminogen activator
