# Supplementary material for: Plasmin-Induced Activation of Human Platelets Is Modulated by Thrombospondin-1, Bona Fide Misfolded Proteins and Thiol Isomerases
Source: Int J Mol Sci. 2020 Nov 23;21(22):8851. doi: 10.3390/ijms21228851 (PMC7700677; doi:10.3390/ijms21228851)
Supplement: Supplementary file 1 [file ijms-21-08851-s001.pdf]

Supplementary Figures

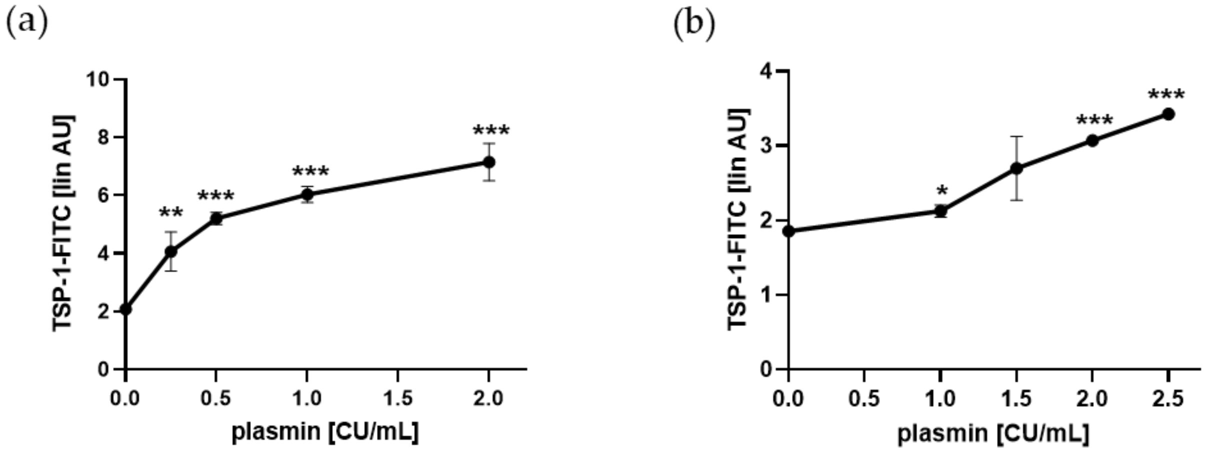

Figure S1. Plasmin induces TSP-1 binding to human platelets. Human platelets, gel-filtered (***a***) or in PRP (***b***) were treated with increasing concentrations of plasmin for 5 min at RT and labeled with TSP-1-FITC. The linear median fluorescence intensity of gated platelets was analyzed and presented as arbitrary units (AU). Data are represented as means ± SD from three independent experiments. * $p < 0.05$, ** $p < 0.01$, *** $p < 0.001$ versus vehicle control without plasmin.

(a)

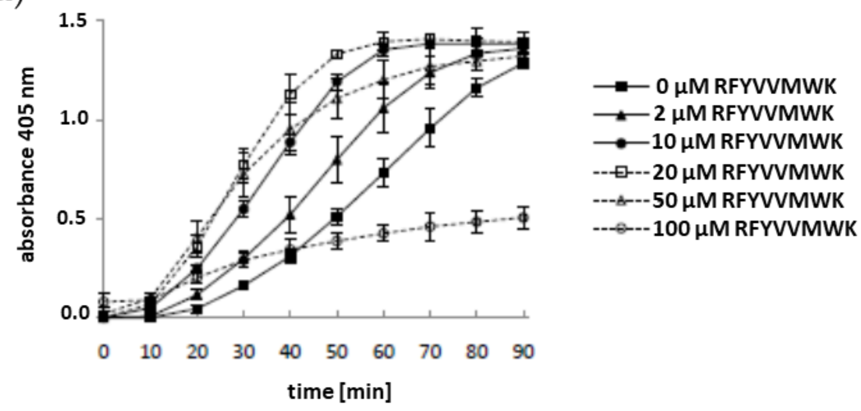

(b)

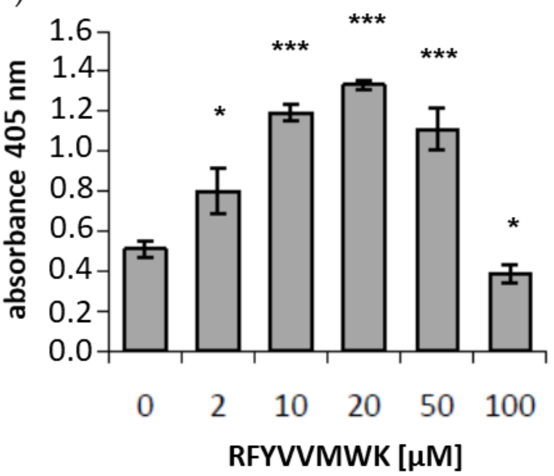

Figure S2. Effect of the TSP-1 peptide RFYVVMWK on tPA-mediated plasminogen conversion to plasmin. RFYVVMWK was pre-incubated with 0.5 µg/mL tPA for 20 min at room temperature in PBS, pH 7.4. The reaction was started by the addition of 50 µg/ml plasminogen and 600 µM plasmin substrate S-2403. Plasmin activity was recorded by the change in absorbance at 405 nm over 90 min at 37 °C. tPA, plasminogen and substrate without RFYVVMWK served as reference control. (***a***) Representative plasmin activity kinetics. (***b***) Quantitative data of absorbance at 405 nm determined after 50 min reaction time at 37 °C. Data are presented as means ± SD from three independent experiments. * $p < 0.05$, *** $p < 0.001$ versus reference control without RFYVVMWK.

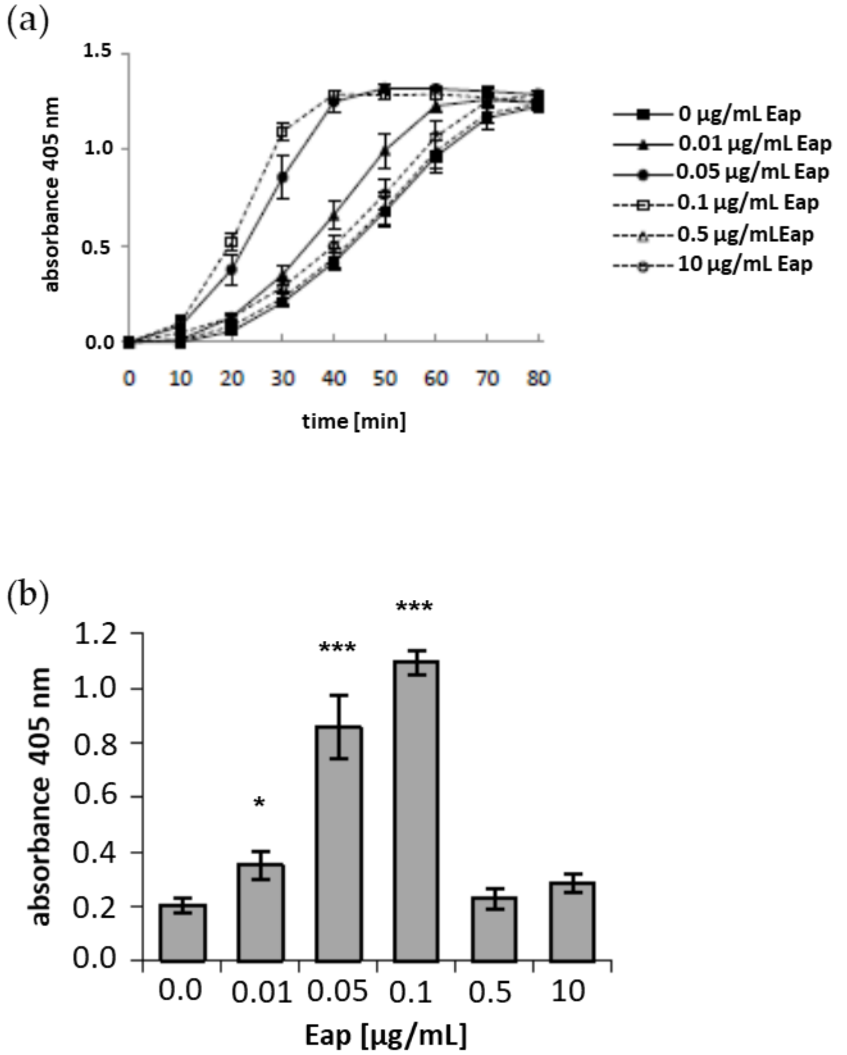

Figure S3. Effect of the staphylococcal extracellular adherence protein (Eap) on tPA-mediated plasminogen conversion to plasmin. Eap was pre-incubated with 0.5 μg/mL tPA for 20 min at room temperature in PBS, pH 7.4. The reaction was started by the addition of 50 μg/mL plasminogen and 600 μM plasmin substrate S-2403. Plasmin activity was recorded by the change in absorbance at 405 nm over 80 min at 37 °C. tPA, plasminogen and substrate without Eap served as reference control. (***a***) Representative plasmin activity kinetics. (***b***) Quantitative data of absorbance at 405 nm determined after 30 min reaction time at 37°C. Data are presented as means ± SD from three independent experiments. * $p < 0.05$, *** $p < 0.001$ versus reference control without Eap.

(a)

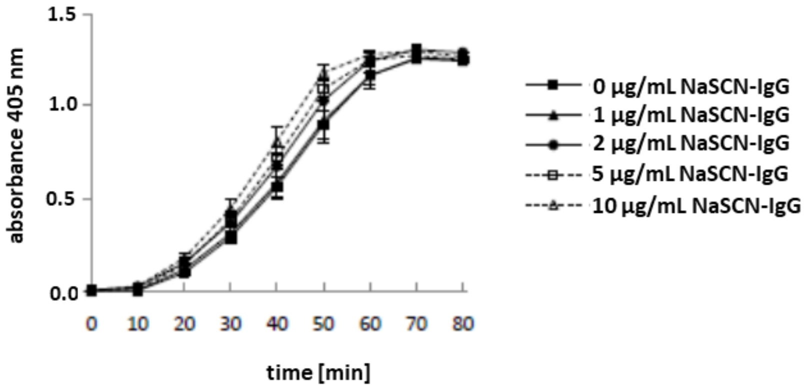

(b)

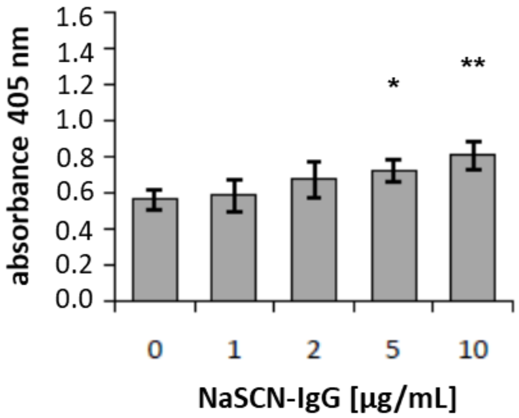

Figure S4. Effect of NaSCN-denatured IgG (NaSCN-IgG) on tPA-mediated plasminogen conversion to plasmin. NaSCN-IgG was pre-incubated with 0.5 µg/mL tPA for 20 min at room temperature in PBS, pH 7.4. The reaction was started by the addition of 50 µg/mL plasminogen and 600 µM plasmin substrate S-2403. Plasmin activity was recorded by the change in absorbance at 405 nm over 80 min at 37 °C. tPA, plasminogen and substrate without NaSCN-IgG served as reference control. (***a***) Representative plasmin activity kinetics. (***b***) Quantitative data of absorbance at 405 nm determined after 40 min reaction time at 37°C. Data are presented as means ± SD from three independent experiments. * $p < 0.05$, *** $p < 0.001$ versus reference control without NaSCN-IgG.
